# Adult Intussusception Secondary to Inflammatory Fibroid Polyp

**DOI:** 10.5811/westjem.2015.4.26399

**Published:** 2015-06-23

**Authors:** Nobuhiko Kimura, Michael Hight, James Liang, Ronald Willy, Kimberly Liang, Jacob Camp

**Affiliations:** Naval Hospital Okinawa, Emergency Department, Okinawa, Japan

A 30-year-old man presented to the emergency department for two weeks of diffuse abdominal pain and an episode of emesis. He denied fever, prior surgery, or any other illnesses. The patient reported going on a “crash diet regimen” one month prior, resulting in an intentional weight loss of 25lbs in 30 days. He also reported two episodes of melena-type bowel movements prior and had an esophagogastroduodenoscopy eight days earlier, which was noted to be normal. On physical examination he was mildly ill-appearing with diffuse abdominal tenderness without peritoneal signs. Computed tomography of his abdomen and pelvis showed a small bowel obstruction in the jejunum. A diagnostic laparoscopy was performed. Operative findings revealed 2.5cm lesion at distal portion of thickened small bowel and intussusception 10–12cm proximal to this. He underwent laparotomy with small bowel resection. Pathological examination of the specimen revealed a 4.0cm inflammatory fibroid polyp.

Intussusception is rare in adults, accounting for 5% of all cases of intussusceptions and 5% of bowel obstructions in adults (Figure 1 and Figure 2).^1^ Approximately 90% of cases of intussusception in adults are secondary to a pathologic condition that serves as a lead point for the intussusception, such as carcinomas, polyps, or Meckel’s diverticulum, etc.^2^

Inflammatory fibroid polyps (IFPs) are rare, benign tumors that can arise throughout the gastrointestinal tract.^3^ The most common site is the gastric antrum (66–75%), followed by the small bowel (18–20%) and colorectal region (4–7%).^4^ Gastric and colon IFPs are typically identified incidentally, whereas small intestinal lesions can present with chronic abdominal pain, lower gastrointestinal bleeding, anemia and rarely small bowel obstruction due to intussusception.^5^ Although IFPs are rare and benign conditions, surgery is the only solution in case of bowel obstruction.^4^ The patient’s postoperative course was unremarkable, and he was discharged on postoperative day 4.

## Figures and Tables

**Figure 1:**
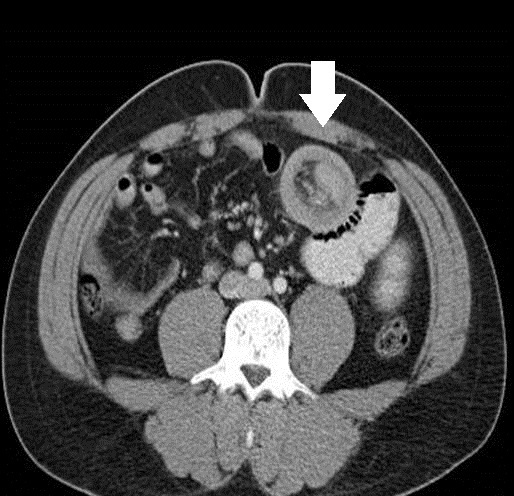
Axial image-donut sign (arrow) indicative of intussusception: fat, vessels and a segment of small bowel (the intussusceptum) prolapsed into the lumen of another segment of small bowel (the intussuscipiens, the outer ring or donut).

**Figure 2:**
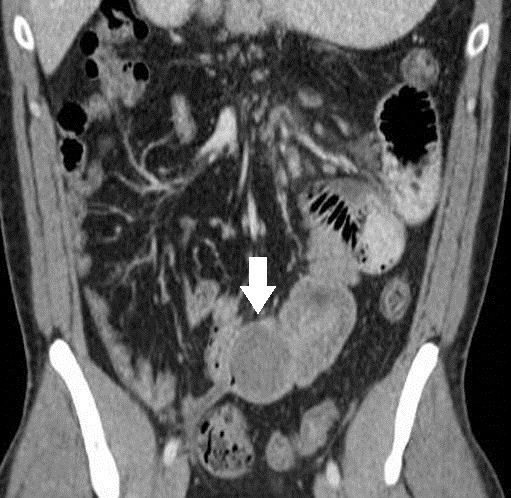
Coronal image: 4cm benign inflammatory polyp (arrow) at the distal intussusceptum, the lesion that served as the lead point for the intussusception.
